# Moving Thailand’s mountain of alcohol-related harm 

**DOI:** 10.2471/BLT.17.020717

**Published:** 2017-07-01

## Abstract

Few low- and middle-income countries have done so much to protect their citizens from alcohol-related harms, but Thailand still faces challenges. Apiradee Treerutkuarkul reports.

Kumron Choodecha recalls how a spontaneous grassroots movement sprung up overnight to oppose the influence of the alcohol industry in his country.

Kumron was one of thousands of people who took to the streets of the capital Bangkok in 2005 to protest against the application for a stock market listing by the country’s biggest brewer and distiller. They succeeded and Thai Beverages' submission was rejected.

As a music and sports event organizer in the 1990s, Kumron had witnessed first-hand the dire social and health effects of alcohol on teenagers and young people.

“Some drank so much they fell out with their families, did badly at school, had accidents and injuries, got into fights and ended up in trouble with the police,” Kumron recalls.

Kumron runs Alcohol Watch, a nongovernmental organization that campaigns with civil society groups in Thailand for stronger alcohol control policies and action, and plays a crucial role in gathering support especially among young people.

This grassroots movement is one the three cornerstones of the country’s alcohol control policy that Thais call the “triangle that moves the mountain”. A strong scientific community providing evidence on the harms of alcohol and robust policies based on that evidence make up the other two corners. 

Nearly 13 million people – about one fifth of the Thai population – signed a petition in support of Thailand’s Alcohol Beverage Control Act when it was first proposed.

The law, which was passed in 2008, bans direct advertising and promotion of alcoholic beverages and sets the minimum age for drinking alcohol at 18 years. It requires these products to carry warnings that alcohol sales to anyone under 20 years of age is prohibited and that drinking can reduce the ability to drive. The law also restricts alcohol sales to certain hours and days.

In 2009 the Thai National Health Assembly adopted a national strategic plan on alcohol control to bring together all sectors to tackle alcohol-related harms.

Today Thailand is one of the few developing countries with laws and policies aimed at preventing alcohol-related problems, such as liver disease, cancers and alcohol dependence as well as road crashes, poverty, violence and crime. 

“Thailand is an example of a developing country that has taken tough action domestically and at the same time contributed to regional and global action,” says Dag Rekve from the Department of Mental Health and Substance Abuse at the World Health Organization (WHO) in Geneva.

In spite of all the progress the country has made, it faces major challenges. 

Thailand has the highest alcohol per capita consumption compared with other countries in WHO’s South-East Asian region and is above the global average of 6.4 litres of pure alcohol per year with 7.2 litres among those aged 15 years and over.

Alcohol consumption is one of four leading health risk factors in Thailand, along with tobacco, diet and hypertension, according to the Global Burden of Disease project.

There are growing concerns about the negative effects of alcohol consumption on the lives of others, through drink-driving, intimate partner and other kinds of violence, and alcohol consumption during pregnancy which may lead to birth defects.

Orratai Waleewong, a researcher at the International Health Policy Program in Thailand, started studying the secondary effects of alcohol consumption a few years ago after reading that these problems were under-researched. 

One study she published in 2015 found that 80% of survey respondents suffered from the adverse effects of other peoples’ drinking in some way. “We still don’t know the full extent of these secondary effects of alcohol consumption in Thailand, as many of these problems are veiled by shame, fear, taboos and other social norms,” she says.

“Many of these problems are veiled by shame, fear, taboos and other social norms.”

Orratai Waleewong

“Data on alcohol-related harm to others will also provide us with a better understanding of who is likely to be affected and what programmes and response services are needed to prevent these problems,” she says.

The Thai Health Promotion Foundation (ThaiHealth) is a core part of Thailand’s alcohol-control armament. Set up in 2001, it uses alcohol and tobacco tax revenues to run campaigns and fund research.

For Bundit Sornpaisarn, a veteran alcohol and tobacco researcher, two key challenges facing him as director of the Major Risk Factors Control section at ThaiHealth, are how to prevent young people from starting to drink alcohol and how to reduce consumption overall.

“Alcohol consumption has been increasing in our country since the 1980s and, unless we take more tough action, it will increase further among the youth and among women – groups where abstinence has been high traditionally,” Bundit says.

To curb drinking initiation while reducing overall consumption, Bundit says Thailand plans to introduce a new excise tax system this year to raise the price of alcoholic beverages. 

Such measures are accompanied by ThaiHealth social marketing campaigns including television and radio spots on alcohol-related harms, such as drink-driving, domestic violence and the risks of unprotected sex, including teenage pregnancies and HIV infection.

“Most Thais – about 95% of us – are Buddhists. According to religious precepts, Thais did not drink alcohol traditionally but in spite of that, alcohol consumption in our country has increased in recent decades,” says Bundit. 

Some campaigns focus on Buddhist traditions, for example one promotes an alcohol-free Buddhist Lent – the annual period for fasting and reflection – while others restrict alcohol sales during national festivities such as the Songkran Thai New Year.

The 2008 law bans marketing of alcoholic beverages in mainstream media, but does not cover some sponsorship, such as on-site promotion, or digital media such as websites, social media, mobile phone applications and product placement in film and television.

“Social media is the new tool for the alcohol industry to market their products to teenagers and young adults,” says Dr Samarn Futrakul, the former director of Thailand’s Office of Alcohol Control Committee who now works on alcohol control in the health ministry, referring to Thai celebrities who post their pictures drinking brand-name alcoholic beverages.

An image of a Thai actor pouring beer from a clearly branded bottle with the caption “let the party begin”, recently went viral on social media triggering a public debate in Thailand over alcohol control and calls to close loopholes in the law.

Concerned about interference from the alcohol industry Samarn says: “Our efforts to develop a strong alcohol control policy are being hampered by the involvement of the private sector, including the alcohol industry, in the policy-making process”.

Kumron agrees. He believes this is one reason why the national alcohol committee, set up as part of the country’s 2009 alcohol control strategy, has not been active recently.

“We want the authorities to strictly enforce existing laws on alcohol, such as zoning and taxation to curb consumption. But we can’t achieve much when the alcohol company executive sits in the national policy-making body,” Kumron says. Zoning refers to banning alcohol sales from the area around schools and colleges. 

Thailand has not won all its battles to control alcohol. Inspired by warnings on cigarette-packet labelling in many countries, a bill requiring alcohol companies to include pictures on bottles of alcohol highlighting alcohol-related social and health harms, was rejected in 2010 on the basis of international trade agreements. 

“Thailand is fighting the alcohol problem on two fronts: against the local alcohol industry and against the transnational alcohol companies,” Bundit says.

“Thailand is fighting the alcohol problem on two fronts: against the local alcohol industry and against the transnational alcohol companies.”

Bundit Sornpaisarn

Three companies account for 92% of the Thai alcoholic beverages market, but there is a rapid expansion by transnational alcohol corporations into Thailand and other developing countries with high abstention rates, youthful populations and growing economies.

Thailand is not the only country facing battles to control alcohol use in the national and global arena. In 2010, the World Health Assembly endorsed the WHO *Global strategy to reduce harmful use of alcohol *to support countries in their efforts to reduce alcohol-related health and social problems. 

“Since then considerable progress has been made worldwide in implementing the *Global strategy to reduce harmful use of alcohol*, though mainly in wealthy countries,” says Rekve.

“Less progress has been made in low- and middle-income countries, where alcohol consumption and problems related to alcohol are on the rise,” says Rekve, noting that many of these countries still lack sufficient political commitment and a national alcohol policy; important first steps towards effective control. 

The WHO global strategy calls for political commitment in countries and recommends a range of policies and measures to reduce alcohol-related harm, including pricing, taxation, drink-driving sanctions, bans on marketing and sponsorship as well as providing support for community awareness programmes and monitoring and surveillance.

In 2012, the *Global action plan for the prevention and control of noncommunicable diseases 2013–2010* set a target for countries to reduce alcohol consumption by 10%. 

In addition, sustainable development target 3.5 is to strengthen the prevention and treatment of substance abuse, including narcotic drug abuse and harmful use of alcohol. 

“Our experience in Thailand shows just how much can be achieved with the right mix of grassroots support, political commitment and scientific evidence,” says Orratai, adding: “Knowledge about the harms caused by drinking alcohol both to the drinkers and the people around them shows us the path to take and how far we have to go.” 

**Figure:**
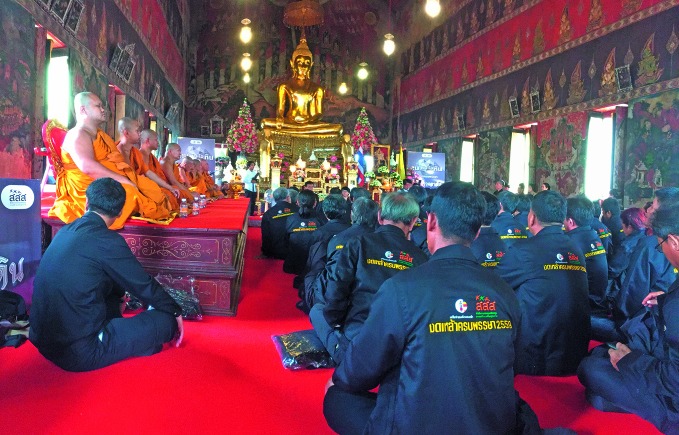
Buddhists who refrained from drinking alcohol during a three-month alcohol-free Buddhist Lent campaign in 2016, worship in a temple.

Courtesy of ThaiHealth

**Figure:**
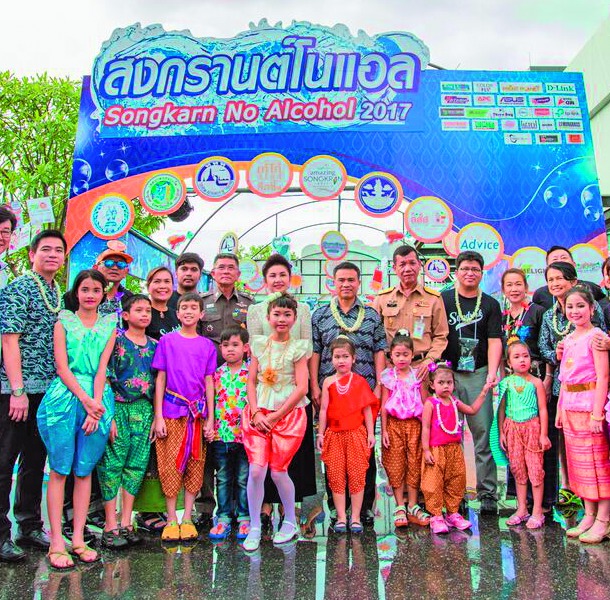
Celebrating an alcohol-free Songkran Thai New Year in April 2017.

Courtesy of the StopDrink Network Thailand

